# Supplementary material for: Effects of Trichoderma harzianum combined with Phanerochaete chrysosporium on lignin degradation and humification during chicken manure and rice husk composting
Source: Front Microbiol. 2025 Feb 28;16:1515931. doi: 10.3389/fmicb.2025.1515931 (PMC11906335; doi:10.3389/fmicb.2025.1515931)
Supplement: Supplementary file 1 [file Data_Sheet_1.zip › 6.Function/All/FunGuild/l1/heatmap_group.pdf]

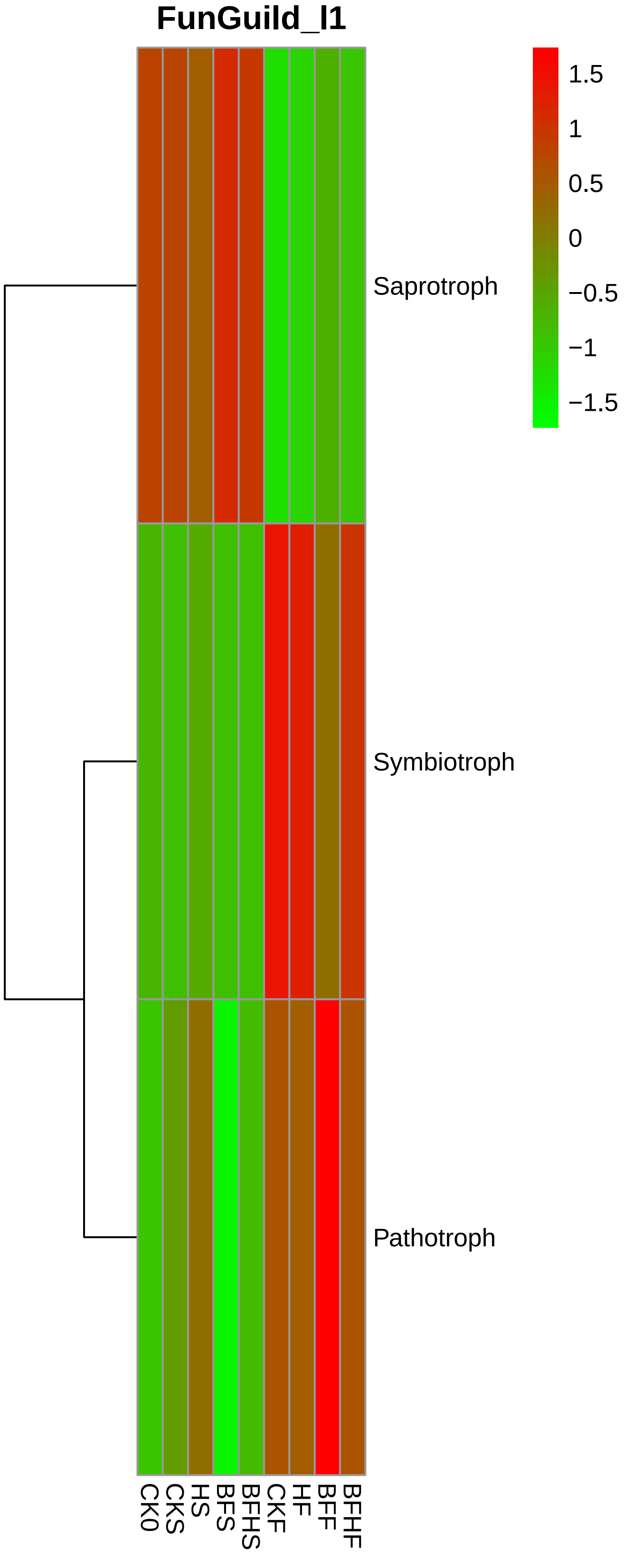

FunGuild_l1
Saprotroph
Symbiotroph
Pathotroph
CK0
CKS
HS
BFS
BFHS
CKF
HF
BFF
BFHF
1.5
1
0.5
0
−0.5
−1
−1.5
